# EBV genome analysis in multiple sclerosis shows extensive viral diversity and links to autoimmunity

**DOI:** 10.1093/narmme/ugag019

**Published:** 2026-03-24

**Authors:** Joonas Lehikoinen, Leo Hannolainen, Osma S Rautila, Zachery W Dickson, Lari Pyöriä, Liane Dieterich, Lilja Jansson, Silja Tammi, Klaus Hedman, Maria F Perdomo, Pentti J Tienari

**Affiliations:** Translational Immunology Research Program, University of Helsinki, Helsinki, P.O 63, 00014, Finland; Department of Neurology, Neurocenter, Helsinki University Hospital, Helsinki, 00290, Finland; Department of Virology, Medicum, Faculty of Medicine, University of Helsinki, Helsinki, 00290, Finland; Translational Immunology Research Program, University of Helsinki, Helsinki, P.O 63, 00014, Finland; Department of Virology, Medicum, Faculty of Medicine, University of Helsinki, Helsinki, 00290, Finland; Department of Virology, Medicum, Faculty of Medicine, University of Helsinki, Helsinki, 00290, Finland; Translational Immunology Research Program, University of Helsinki, Helsinki, P.O 63, 00014, Finland; Rudolf Virchow Center, Biomedicine, University of Würzburg, Würzburg,97080, Germany; Translational Immunology Research Program, University of Helsinki, Helsinki, P.O 63, 00014, Finland; Department of Neurology, Neurocenter, Helsinki University Hospital, Helsinki, 00290, Finland; Research and Development, Finnish Red Cross Blood Service, Helsinki, 01730, Finland; Department of Virology, Medicum, Faculty of Medicine, University of Helsinki, Helsinki, 00290, Finland; Department of Virology, Medicum, Faculty of Medicine, University of Helsinki, Helsinki, 00290, Finland; Translational Immunology Research Program, University of Helsinki, Helsinki, P.O 63, 00014, Finland; Department of Neurology, Neurocenter, Helsinki University Hospital, Helsinki, 00290, Finland

## Abstract

Epstein–Barr virus (EBV) is a major risk factor for multiple sclerosis (MS), yet the contribution of specific viral variants remains unclear. Complete EBV genome analysis of wild-type variants has not been performed previously. In a pilot-study, targeted EBV sequencing of B cells did not yield adequate coverages due to excess of human DNA. Thus, we developed an *ex vivo* 6-day leukocyte culture method to enrich EBV DNA into supernatant by stimulation with tetradecanoyl phorbol acetate. Using this method in 20 MS patients and 20 controls, we obtained near-complete EBV genomes in 9 MS patients (average coverage 97%) and in 4 controls (average 85%). We identified 1088 single-nucleotide variants (33% missense variants) outside repetitive regions that met stringent quality criteria. Overall, 94% of the variants were present in three or more subjects, thus unlikely generated during the culture and several were exclusive to MS, worth of further investigation. *In silico* analysis indicated that variants within the EBNA1 cross-reactive region change human leukocyte antigen (HLA) peptide binding affinity, suggesting altered antigen presentation. These results illustrate a method for enriching EBV genomes and present the first wild-type sequences from MS patients. The observed diversity highlights potential for future studies in EBV-associated diseases and vaccine development.

## Graphical Abstract

**Graphical Abstract:**
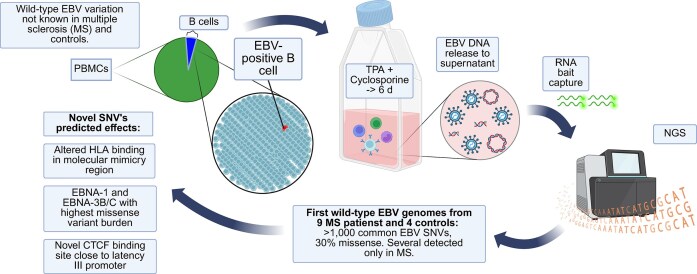

## Introduction

Multiple sclerosis (MS) is the most common demyelinating inflammatory disease of the central nervous system, affecting almost 3 million people worldwide. Epstein–Barr virus (EBV) has recently been identified as a major risk factor for MS [1–3]. Among the key unresolved questions is, how such a common infection (worldwide EBV seropositivity > 90% in adults) contributes to a disease as relatively rare as MS (0.2% prevalence in high-risk countries).

EBV is an oncovirus with sequence variation shown to contribute to carcinogenesis [4]. In certain lymphomas, a deletion leads to strong antiapoptotic effects in the host cell [5]. In nasopharyngeal carcinoma, which is endemic in South China, a specific variant in the *BALF2* gene associates with this cancer [6].

The role of EBV sequence variation in MS is unclear. EBNA1 is of particular interest for genomic analysis, as several of its peptides share sequence similarities with human proteins implicated in molecular mimicry in MS, including anoctamin 2 (ANO2), glial cell adhesion molecule (GlialCAM), alpha B crystallin (CRYAB), and myelin basic protein (MBP) [7–13]. Previously, EBV variants have been examined by polymerase chain reaction (PCR)-based studies, and a few variants in *EBNA1, EBNA-2, BRRF2*, and *LMP-1* have been reported to associate with MS [14–16]. While the findings in EBNA-2 have been replicated [17], others have found no differences [18].

Thus far, EBV genomes have been derived from malignant cell lines (e.g. Burkitt lymphomas, Hodgkin lymphomas, nasopharyngeal carcinomas and gastric carcinomas) or from *in vitro* generated lymphoblastoid cell lines (LCLs), in which the viral loads are high [19]. Already in 1979, lymphocytes from patients with active MS were reported to have an increased tendency for spontaneous LCL transformation compared to controls or patients with stable MS [20]. This observation aligns with recent studies finding signs of unstable EBV latency in MS patients’ brain samples [21] and transcriptomes of B cells from MS patients’ cervical lymph nodes [22]. Soldan *et al.* showed that spontaneous LCLs from MS patients produce higher amounts of inflammatory cytokines relative to controls and suggested that unstable EBV latency might drive immune activation in MS [23]. In their study, complete EBV genomes were recovered after prolonged LCL culture from seven MS patients and two controls. However, extended culture periods—often exceeding 20 weeks—may result in clonal selection and accumulation of *de novo* mutations.

Building on our previous findings indicating that EBV is more prevalent in B cells than previously recognized [24] (on average 1 in 2000 B cells), we sought to characterize the viral genomes in both MS patients and controls. Utilizing our established next-generation sequencing (NGS) pipeline, which has successfully recovered complete genomes of relatively rare viruses from various tissue samples [25, 26], we designed a custom panel of EBV biotinylated RNA baits to perform targeted sequencing on DNA samples from CD19^+^ B cells. This pilot study laid the groundwork for a novel 6-day peripheral blood mononuclear cell (PBMC) culture method enabling enrichment of EBV genomes. The whole study aimed to generate high-coverage EBV genome sequences directly from *ex vivo* cells, providing a foundation for comparative analyses between MS patients and controls and contributing to deeper understanding of EBV genomic diversity.

## Materials and methods

### Subjects

For direct analysis of CD19^+^ cells we collected venous blood samples from 30 newly diagnosed MS patients and 30 controls, who were volunteers, or had other neurological symptoms or diseases. For PBMC culturing and endogenous EBV reactivation we collected venous blood samples from 20 newly diagnosed MS patients and 20 healthy volunteers from Helsinki University Hospital neurology outpatient clinic. Demographic characteristics of the patients and controls of both cohorts are shown in Table 1. Individual level characteristics are summarized in Supplementary Table S1.

**Table 1.** Demographic and laboratory data of MS patients and controls

*Ex vivo*CD19^+^cells	MS patients (*n* = 30)	Controls (*n* = 30)
Age, mean/median [SD]	33.2/32.5 [8.5]	38.3/39 [10.1]
Sex, female, *n* (%)	20 (67%)	26 (87%)
HLADRB1*15:01, n (%)	12 (40%)	7 (23%)
EBV Ab IgG, mean/median [SD]	159.3/170 [34.5]	131.0/125 [47.3]
CMV Ab IgG, mean/median [SD]	100.7/71.5 [101.0]	55.7/0 [75.5]
CSF Oligoclonal bands, *n* (%)	30 (100%)	-
Barkhof MRI criteria, mean/median [SD]	3.7/4 [0.71]	-
EDSS, mean/median [SD]	1.6/1.8 [1.2]	-
**Cultured and stimulated PBMCs**	**MS patients (*n* = 20)**	**Controls (*n* = 20)**
Age, mean/median [SD]	35.1/35 [5.4]	34.1/32 [8.8]
Sex, female, *n* (%)	14 (70%)	15 (75%)
HLADRB1*15:01, *n* (%)	8 (40%)	7 (35%)
EBV Ab IgG, mean/median [SD]	143.2/165 [53.4]	128.9/140 [46.5]
CMV Ab IgG, mean/median [SD]	79.6/18.5 [101.2]	81.7/44 [94.9]
CSF Oligoclonal bands, n (%)	20 (100%)	-
Barkhof MRI criteria, mean/median [SD]	3.4/4 [0.82]	-
EDSS, mean/median [SD]	1.2/1.0 [0.88]	-
**Cultured and stimulated PBMCs (subjects with highest EBV DNA yield)**	**MS patients (*n* = 9)**	**Controls (*n* = 4)**
Age, mean/median [SD]	35.9/36 [5.4]	28.8/29.5 [5.4]
Sex, female, *n* (%)	5 (56%)	3 (75%)
HLADRB1*15:01, *n* (%)	4 (44%)	2 (50%)
EBV Ab IgG, mean/median [SD]	171.7/180 [38.6]	148.3/165 [62.6]
CMV Ab IgG, mean/median [SD]	84.4/37 [106.9]	22/0 [44]
CSF Oligoclonal bands, *n* (%)	9 (100%)	-
Barkhof MRI criteria, mean/median [SD]	3.6/4 [0.73]	-
EDSS, mean/median [SD]	1.0/1.0 [0.90]	-

All MS patients fulfilled the McDonald 2017 diagnostic criteria, and they were all positive for cerebrospinal fluid (CSF) specific oligoclonal bands. Patients were sampled at diagnosis, prior to initiation of any disease modifying treatment. Subjects were matched by Finnish ethnicity. The 20 patients and 20 controls selected for PBMC culturing were additionally matched by age, sex, and HLA-DRB1*15:01.

### Ethics approval and consent to participate

This study was approved by the ethical committee of the Helsinki University Hospital (Dno 83/13/03/01/2013) and the patients and controls signed a written informed consent for the study according to the Declaration of Helsinki.

### Extraction of *ex vivo* CD19^+^ cells’ DNA

We separated PBMCs with Ficoll-Paque™ PLUS (Cytiva, USA), and collected CD19^+^ cells using magnetic beads (CD19 MicroBeads, Miltenyi Biotec, USA) and extracted their DNA with InviTrap^®^ Spin Universal RNA Mini Kit (Stratec Biomedical Systems, Germany) according to the manufacturers’ instructions.

### Serology analysis

Plasma was analysed in an accredited laboratory (HUS Diagnostic Center, Helsinki, Finland) for EBV viral capsid antigen (VCA) antibody levels with a quantitative method (Euroimmun), and cytomegalovirus (CMV; whole virus lysate, strain AD169; semiquantitative method; Abbott Alinity) IgG antibody levels to confirm subjects serostatus. We additionally performed immunoblotting with recomLine EBV IgG [Avidität][IgA] (Mikrogen Diagnostik, Germany) according to the manufacturer’s instructions to assess antibody positivity against six different EBV proteins.

### Cell culturing

We modified classic LCL-generation protocols [27] and developed a novel culturing method in order to enrich endogenous EBV. We collected 70 ml of venous blood into EDTA tubes and separated PBMCs with Ficoll-Paque™ PLUS (Cytiva, USA). We resuspended the cells into 1 ml of pure PBS and counted the cells using Trypan-Blue and Bio-Rad TC20 Automated Cell Counter.

After counting, we seeded the cells into culture media at ∼2 × 10^6^ cells/ml. We used a mix of Roswell Park Memorial Institute (RPMI) 1640 with 10% fetal bovine serum (FBS), 2 mM L-glutamate, 100 IU/ml penicillin, 100 µg/ml streptomycin, and 0.5 µg/ml amphotericin-B as basis culture media (BCM). Incubations were at 37°C temperature and 5% CO_2_.

During the first cultures, each day 2 ml of supernatant was collected and stored at −20°C for quantitative PCR (qPCR) as a way of monitoring the virus secretion. We confirmed EBV DNA in the supernatant during days 5 and 6 of the culture.

First incubation lasted for 48 h, after which we collected the cells by centrifuging at 300 × *g* for 10 min. We then resuspended the cells into BCM with added 0.01 µg/ml of cyclosporin-A and incubated them for 1 h in order to hinder EBV recognizing T cells from activating during the stimulation. Then, we added tetradecanoyl phorbol acetate (TPA), a potent mitogen used routinely to induce EBV’s lytic cycle [28], to 20 ng/ml concentration for 1 h of incubation. After this, we washed the cells two times by centrifuging at 300 × *g* for 10 min and resuspending with BCM. Finally, we resuspended the cells into BCM with 0.005 µg/ml of cyclosporin-A and incubated for 96 h. Following the final incubation, we centrifuged the cells at 600 × *g* for 10 min at 4°C, collected the cells and froze them with FBS and 10% dimethyl sulfoxide. We collected the supernatant and filtered it through a 0.45-μm filter slowly. Then we performed 10 kDa filter Falcon tube centrifugation at 3000 × *g* for 10–30 min to further concentrate the supernatant, which we thereafter stored frozen at −80°C.

### Extraction of cell cultures

Before extraction, ∼30 ml of cultured supernatants were concentrated using Amicon ultra 10KDa filters by centrifugation at 3000 × *g* for 10 min to a final volume of 3 ml.

The concentrated supernatants were extracted using the QIAamp Circulating Nucleic acid (cat. no./ID: 55114) according to the 5 ml serum protocol specified by the manufacturer. The final elution volume was in 110 µl of AVE buffer. A 3 ml high grade water sample was included as negative control in the extractions and in downstream experiments.

### Quantitative PCR

The DNA extracts of the cultured supernatants of MS patients and controls were screened for EBV and human *RNase P* single copy gene [29] by qPCR, as described [30]. Both qPCRs were performed using the AriaMx Real-Time PCR System (Agilent). The quantities of *RNAse P* were used to normalize the viral DNA to viral copies per one million cells. The sample handling, PCR reactions, plasmids handling and amplifications were executed in separate spaces and hoods to avoid contaminations. Negative controls were included in every PCR run.

### Estimation of EBV-to-human-DNA ratio in *ex vivo* CD19^+^ cells

Assuming that B cells represent 5% of PBMCs, and that on average 0.05% of B cells are EBV-positive [24] and contain around 100 copies of EBV-DNA (0.17 Mb) each [31], and the purity of B cells after separation is 85% [32], we estimated the EBV-DNA to human-DNA ratio to be ≈ 1/883 000 in CD19^+^ cells.

This results from the following calculation: The EBV-DNA amount per B cell equals to 0.17 Mb × 100 copies × 0.0005 × 0.85 = 0.0072 Mb. Diploid human DNA per B cell (average 6350 Mb) and mitochondrial DNA (0.0165 Mb × 400 copies) [33] constitute on average 6357 Mb, which results in EBV-to-human-DNA ratio of 0.0072/6357.

### Next-generation sequencing

We performed EBV targeted enrichment using a custom designed set of biotinylated RNA oligonucleotides (Arbor Biosciences) to maximize capture and coverage in NGS [34]. The baits were 70 nucleotide long and overlapped with three times tiling (∼20 000 baits). We used the following EBV reference sequences for the bait design: DQ279927, KR063344, KP968258, KT273942, MG021316, MH590434, KT273945, MG021307, MH144214, AY961628, KT273946, AJ507799, KR063345, KP968264, KC207813, NC_007 605, NC_009 334, LS992258, KF373730, MH883758.

We fragmented the DNA mechanically with a Covaris E220 to achieve a target length of 200 nucleotides. Then, we prepared the libraries with the KAPA HyperPlus kit (Roche), incorporating unique Dual Index Adapters (Roche). Each sample underwent individual enrichment through two rounds of oligonucleotide hybridization, following the manufacturer’s guidelines for low input DNA using the MyBaits v5 kit (Arbor Biosciences). The libraries were sequenced in NovaSeq X plus 25B (PE151 kit; Illumina).

### NGS data analysis

The sequencing reads were analyzed using a custom pipeline for multi-organ virus analysis, TRACESPipe [25]. Specifically, adapter sequences were removed using Trimmomatic [35] with a maximum mismatch of 2 for a full match. Low-complexity regions were flagged with GTO [25, 36], and reads shorter than 25 bases were discarded. Assembly was performed using an iterative refinement approach combining alignment-based and *de novo* methods. For the alignment-based approach, FALCON-meta [37] was used to select the reference sequence with the highest similarity, followed by read alignment using Bowtie2 [38] with high sensitivity parameters. Duplicate reads were removed, and consensus sequences were generated using SAMtools [39] and BCFtools [40]. *De novo* assembly was carried out using metaSPAdes [41]. We used MAFFT [42] to realign formed consensus sequences to the reference (NC_007605.1), and with Geneious Prime 2024.0.2 (https://geneious.com) we called single nucleotide polymorphisms (SNVs) from bases that had a high sequencing reliability (*P*-value < 10^–6^).

### Genome-wide genotyping and HLA-imputation

DNA from whole blood was genotyped in FIMM (Institute for Molecular Medicine Finland) with Illumina Global Screening Array (GSA)FIN beadchip and genotypes were called with GenomeStudio v.2.0.3 software (Illumina). We used PLINK 1.90 beta (PLINK v1.90b7.2 64-bit (11 December 2023), Shaun Purcell, Christopher Chang, www.cog-genomics.org/plink/1.9/) to conduct additional quality controls. Human leukocyte antigen (HLA) -imputation was carried out at the Finnish Red Cross Blood Service.

High-resolution level alleles of the classical and nonclassical HLA and MIC genes (HLA-A, -B, -C, -DRB1, -DQA1, -DQB1, -DPB1, -DRB3, -DRB4, -DRB5, MICA, MICB, HLA-E, HLA-F, HLA-G and HLA-G 3′UTR and 5′UTR haplotypes) were imputed from the MHC region genotype data using HIBAG v1.38.1 R library [43]. A Finnish HLA reference panel was used [44] for the imputation of the classical HLA and a combined 1000 Genomes and Finnish reference panel was used for the imputation of the nonclassical HLA/MIC [45].

### Multiplex PCR

We designed 41 primers to target 21 high-interest EBV SNVs. Details of the primers are provided in Supplementary Table S2. We used LongAmp^®^  *Taq* Master Mix (New England Biolabs) according to the manufacturer’s instructions. We used 50–1000 ng of extracted DNA from supernatants in two PCR reactions with 5 or 6 primer pairs. We used the following thermocycling protocol: (i) mixture at 95°C for 4 min, (ii) at 95°C for 30 s, (iii) at 65°C for 45 s, (iv) at 72°C for 15 s, steps 2, (iv) repeated for 44 cycles, (v) at 72°C for 7 min, (vi) hold at 4°C. We combined the two multiplex PCR amplicons per patient, and purified them with AMPure XP beads (Beckman Coulter Life Sciences). Finally, we prepared libraries as described above and sequenced them with NovaSeq X plus 25B (PE151 kit; Illumina). We processed the results with TRACESPipe [25], and used Fisher’s exact test to analyze statistical significance.

### Variation burden analysis

We modified the NCBI protocol for Illumina short read sequenced SARS-CoV-2 samples [46]. First, we aligned the reads to NC_007605.1 using Bowtie2. Then, we left-aligned the reads and performed variant calling without filters using GATK (v. 4.5.0) [47]. For quality control in low read depth areas, we included all variants present in at least two different samples or had more than two reads supporting the call in a single sample. The annotation was performed using SNPEff (v. 5.2a) [48] with no LOF annotation and upstream-downstream interval length set to 0. For the final analysis, we only included missense SNVs. The number of SNVs in subjects with MS and controls were compared using a two-tailed *t*-test (excluding sample C1 due to low coverage).

### Haplotype analysis

The haplotype analysis was done using GATK variant calls from the burden analysis, which were first filtered for known repeat regions and indels. After excluding these variants, only positions at which all samples had three or more reads of coverage were included. The C1 sample was excluded due to overall low coverage. After these filters, a haplotype map containing 561 SNVs remained. The average SNV density was one SNV per 306 bp. These haplotypes were then used for phylogenetic tree construction with an UPMGA algorithm from BioPython’s Phylo library (v. 1.85) [49].

### Minor variant analysis

Minor variants (MVs) are positions in the viral genome population that contain two or more nucleotide variants instead of a single nucleotide. MVs were assessed using Lofreq (v.2.1.5) [50] and FreeBayes (v.1.3.6) [51] and only positions called by both methods were considered. These calls were further filtered by excluding sites near homopolymer tracts (over six bases), alleles supported by less than four unique reads or allele frequency <3%.

To assess intra-sample diversity, we calculated Shannon entropy (equation 1) at every site across our 13 sequence libraries and the 9 from Soldan *et al.* [23] and calculated an estimate for the variance for each site, taking into account differences in the sequencing depths. We then used two-sample *t*-test to determine significance between the two groups, see Supplementary Fig. S1.


(1) \begin{eqnarray*}
H\left( P \right) = - \mathop \sum \limits_{i = 1}^{A,\;C,\;T,G} {p_i} \times {\log _2}({p_i})
\end{eqnarray*}

### HLA class II *in silico* binding analysis

We selected the EBNA1 autoantigen cross-reactive region [7–12] with margins: amino acids 347–546. From our NGS data, we identified nonsynonymous variants in this area. Using EBNA1 amino acid (aa) sequence of NC_007605.1 as reference, we fractioned the EBNA1 AA 347–546 sequence into overlapping peptides and introduced the nonsynonymous variants in the mix; 12 aa long peptides were used for HLA class I, and 15 aa long for HLA class II analysis. We used NetMHCII-pan 4.1 (https://services.healthtech.dtu.dk/services/NetMHCIIpan-4.1/) and NetMHC-pan 4.1 (https://services.healthtech.dtu.dk/services/NetMHCpan-4.1/) to analyse *in silico* changes in the HLA class II and HLA class I binding ability respectively to the variant peptides, and compared the results with the reference peptides [52, 53]. We compared the changes in the binding ranks, binding affinity ranks, and HLA class target cores between the variant peptides and the matching reference peptides. Summaries of the analysis are provided in Supplementary Table S3.

## Results

### Pilot study - capture of EBV genome from *ex vivo* CD19^+^ cells’ DNA

We first isolated CD19^+^ cells from PBMCs of 30 MS patients and 30 controls, all EBV seropositive (demographics shown in Table 1).

We performed targeted sequencing using a custom panel of biotinylated RNA baits covering the full EBV genome with triple tiling. The average number of sequencing reads obtained per sample was 175 million (M) (Illumina NovaSeq X plus 25B; range 83 M–935 M). Of these, 0.02% on average mapped to EBV genome (range 0.001%–0.1%). The overall EBV genomic coverages obtained from these cells was relatively low, on average 30% in MS patients (range 0%–95%, average depth 0.9, range 0–5.6) and 24% in controls (range 0%–97%, average depth 0.7, range 0–6.0). Only seven MS patients and five controls had such coverage (>30%) and depth (>1.0) for alignment to be feasible, restricting reliable downstream analysis. We were, however, able to see indications of substantial variation in the EBV genome in comparison to the reference genome. Calling SNVs that occured in at least two subjects, outside repeat regions with variant call quality of ≥20, mapping quality of ≥20 and minimum depth threshold of 3, we found 760 SNVs compared to the reference (NC_007605.1). Due to bias resulting from differences in regional coverages and depths, we did not compare the variant frequencies between patients and controls.

These results indicated that more efficient EBV DNA enrichment was needed to obtain full EBV genomes; the EBV-to-human DNA ratio in this experiment remained too low (estimated ratio ca. 1/900 000, see the ‘Materials and methods’ section).

### Enrichment of EBV in cultured and stimulated PBMCs

In order to enrich the EBV DNA further, we recruited 20 newly diagnosed relapsing MS patients and 20 healthy controls, all EBV seropositive. The subjects were matched by age, sex, and HLA-DRB1*15:01, since this allele has been suggested to facilitate EBV infection [54]. The demographic data are shown in Table 1.

We modified the protocol for EBV stock preparation from EBV-infected B95-8 cells by Hui-Yuen *et al.* [27]. The endogenous EBV was reactivated using TPA (see Supplementary Fig. S2 for an overview and methods for cell culture details). Anti-EBV immune reactions were inhibited by cyclosporin. We tested the PBMC cultures for reactivation of endogenous EBV with *BALF5*-targeted qPCR daily over 1–6 days and detected viral DNA in culture medium from day 5 onwards. Follow-up experiments were carried out using 6-day cultures, in which we detected on average 99 EBV DNA copies/µl in the MS patients and 28 copies/µl in the controls (*P*-value for difference = 0.068, Mann–Whitney U-test). All individual qPCR results are shown in Supplementary Table S1. The higher yield of EBV in MS patients is consistent with previous data demonstrating increased EBV reactivation in MS patients compared to controls [23, 55, 56]. We also measured EBV copies in the uncultured PBMC’s DNA with qPCR, yielding on average 16 EBV DNA copies/µl in the MS patients and 8 copies/µl in the controls. Relative to the cellular fractions, EBV DNA in supernatants, increased by 620% in MS patients and 360% in controls, both being statistically significant (*P-*value = 0.013 and *P*-value = 0.0019, respectively, Mann–Whitney U-test). The culture performed efficiently in samples from both MS patients and healthy controls, showing EBV DNA amplification by real-time PCR in 80% of subjects in each group, with no statistically significant difference between them. After culturing, the supernatants of 2 MS patients and 2 controls remained EBV PCR negative, and were excluded from NGS.

### NGS of the EBV enriched culture supernatants

Upon EBV-targeted sequencing of the culture supernatants of 18 patients and 18 controls, the average number of reads was 68 M (range 17 M–163 M). Of them, on average 0.06% mapped to the EBV genome (range 0.002%–0.5%). The average horizontal coverage was 25% (range 3%–70%). The relatively limited coverage probably resulted from the simultaneous sequencing of uncultured PBMCs (included as controls for *de novo* mutations), which introduced an excess of human DNA into the sequencing run. By comparison of the consensus sequences to the reference genome NC_007605.1, we detected 839 EBV SNVs with >20% frequency and fulfilling quality control with the threshold probability of an incorrect variant call error *P* < 10^–5^. Repetitive regions were excluded from variant calling.

### Analysis of subjects with highest EBV DNA yield

**Figure 1.**
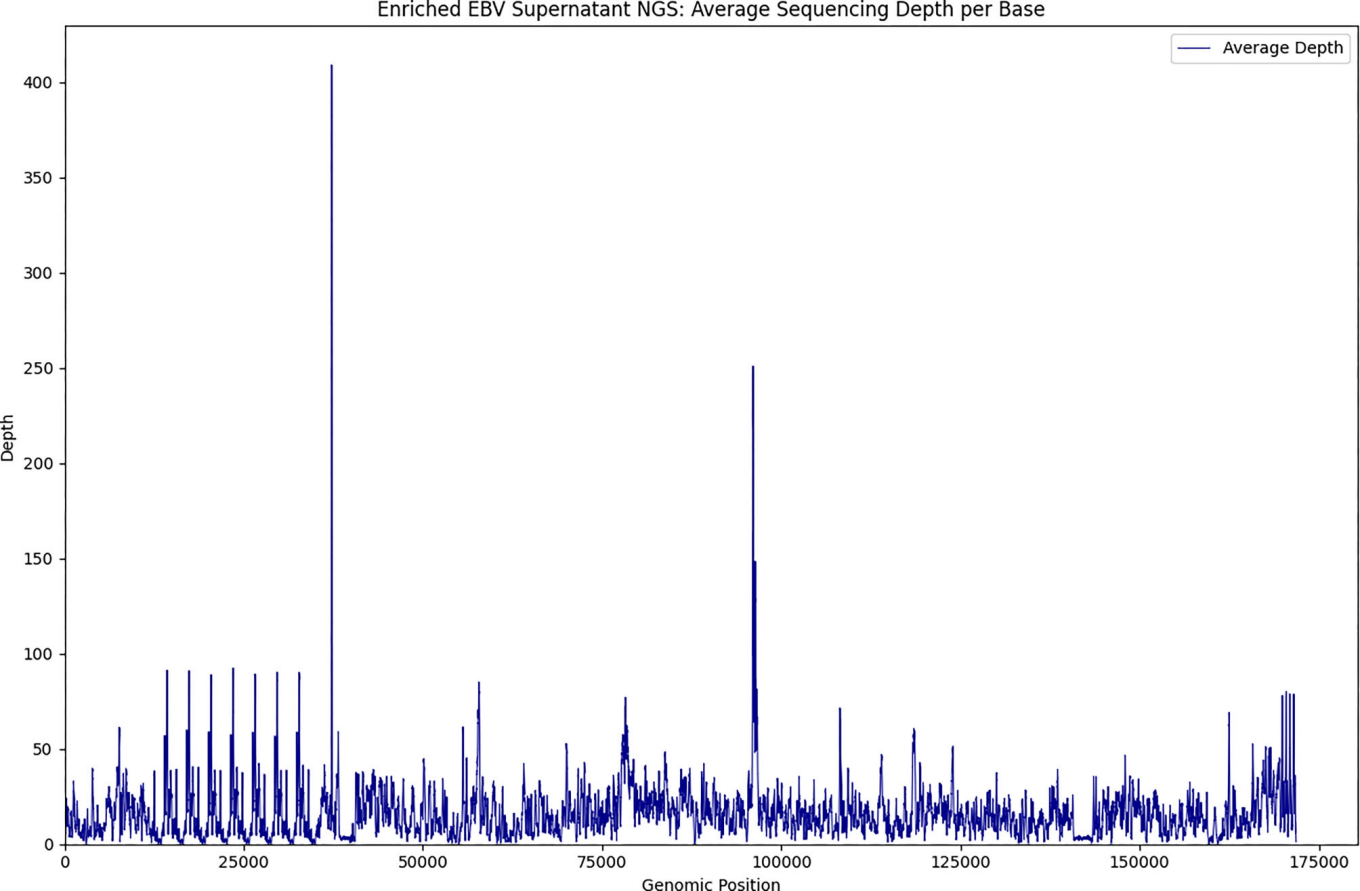
Enriched EBV supernatant NGS: average sequencing depth per base of nine patients and four controls. EBV genomes sequencing depth shows marked variation across regions.

To maximize EBV genome coverage, we selected nine MS patients and four controls (Table 1) with the highest EBV-to-human-DNA ratios in the supernatants. We performed high volume DNA extractions and three parallel libraries in a single sequencing run. We obtained on average 250 M reads (range 141 M–708 M), of which 0.2% on average were mapped to the EBV genome (range 0.01%–1.1%). The average horizontal coverage was 97% (range 90.8%–99.8%) in MS patients and 85% (range 57.4%–97.6%) in controls, and the average depth was 21× (median 17.3, range 5.6–42.2) in MS patients and 7.3× in controls (median 7.2, range 2.5–12.2). The average depth of each genomic position from this run is shown in Fig. 1. The differences between these 13 sequences and reference are illustrated in the SNV distance matrix shown in Supplementary Fig. S3.

From these 13 sequences, we found 1088 SNVs outside repeat regions and 380 SNVs within repeat regions (excluded from the final analysis). Variants were filtered using thresholds of *P* < 10^–6^ for error probability, *P* < 10^–5^ for strand bias, a minimum depth of Three and frequency ≥20%, relative to the reference sequence NC_007605.1. Due to regions of low coverage, 13 SNVs were present in only one of the subjects and 47 in two subjects. The majority of variants (*n *= 1028, 94%) were discovered in three or more subjects. The low number of singleton variants argues against significant amount of random *de novo* variants generated during the cell culture. Notably, 718 of the 839 variants (86%) that were detected in the first NGS of enriched PBMC supernatants, and 576 of the 760 variants (76%) detected in the *ex vivo* CD19^+^ cells were recovered in this subsequent analysis, underscoring the reproducibility of the variant calling. All variants, mutation type, coordinate, gene annotation, and other details are shown in Supplementary Table S4. It is of note that the previously reported, MS-associated EBNA2 1.2 allele [14, 17], representing a combination of eight SNVs, was not found in our data.

**Figure 2.**
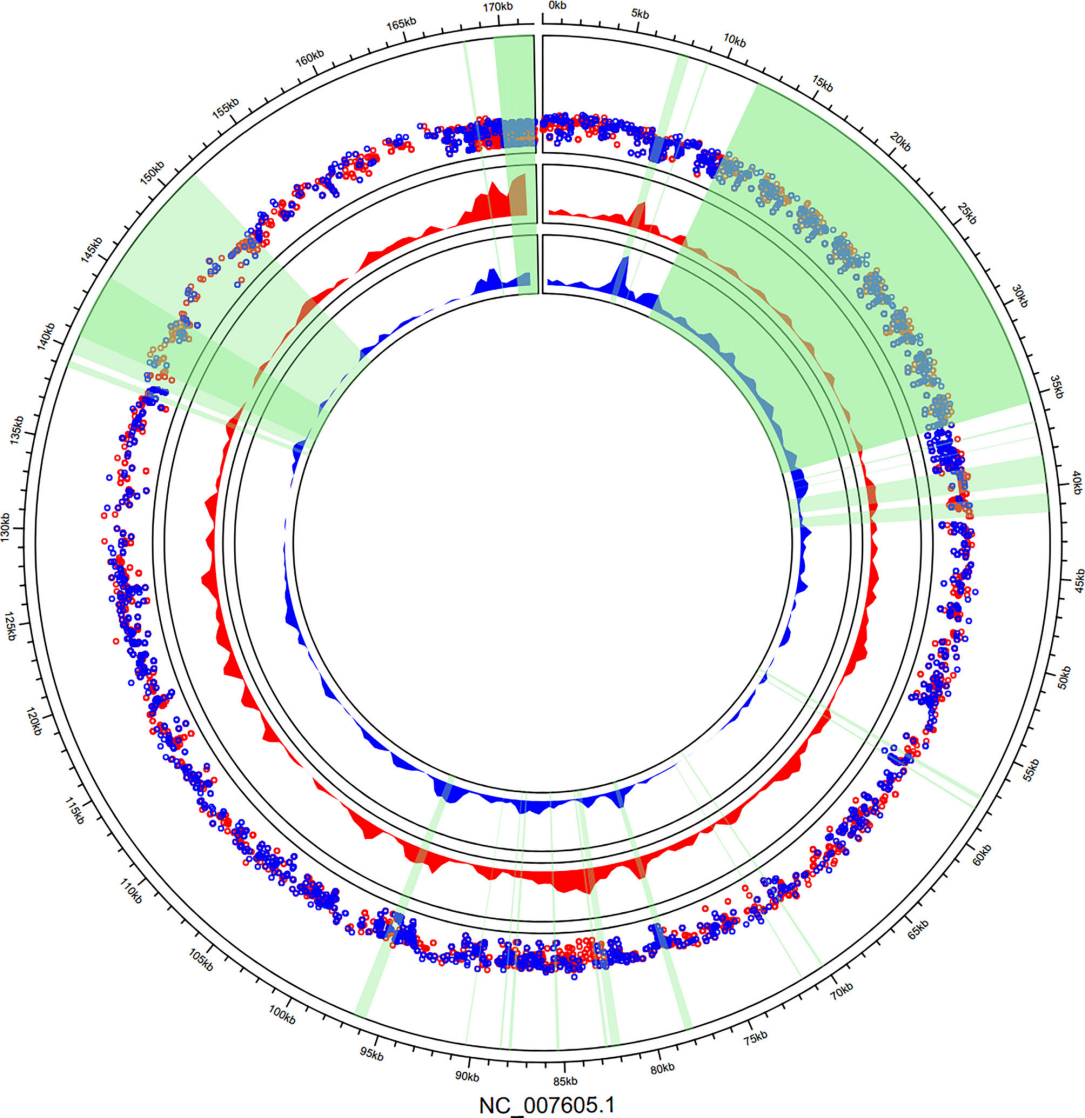
Distribution of EBV allele variants in the EBV genome. Starting from outside, the first ring shows the location of each variant along the coordinates of the reference genome NC_007605.1, the second inner ring shows all variants as a rainfall plot, red dots are MS patient’s SNVs, blue ones are control’s SNVs. Third and fourth inner rings show density plots (red = MS, blue = controls) of all the subjects, illustrating the more varied and more conserved parts of the EBV genome; of note is the visible higher variation density in MS patients as compared to controls in the 85 kb area (EBNA-3B/3C region). Repeat regions are shown in shaded light green, and were excluded in the analysis, including the B95-8 deletion restored from Raji (bp 139 724–151 554).

The distribution of the variants in the EBV genome and density plots are shown in Fig. 2. Of the 1088 SNVs, 355 were nonsynonymous. Among all the 1088 variants, 138 were detected only in MS patients, 8 only in controls, and 153 were shared across all individuals (each variant detailed in Supplementary Table S4). As the MS group was larger and had higher sequencing coverages, the detection of more variants was anticipated. Using a minimum sequencing depth of 5 (with variant call quality ≥20 and mapping quality ≥20), on average, MS-patients had on average, 623 SNVs (range 306–846) and controls 376 (range 81–529). After accounting for the higher coverage and depth of MS-patients, neither the total number of SNVs (average of 547 versus 345 variants at depth 5–50, *P-*value = 0.15, Mann–Whitney U-test) nor number of group-unique variants (average of 33 versus 4 group-unique variants, *P-*value = 0.47, Sequence Kernel Association Test–Optimized [SKAT-O] [57]) differed significantly between MS-patients and controls.

Insertions and deletions (indels) were detected far less frequently than SNVs, and their number did not differ significantly between MS and controls (summary of all variants provided in Supplementary Table S5). One indel (NC_007605.1_168515insA) was found exclusively in MS patients (*n* = 2).

### Variant burden in EBV genes

**Figure 3.**
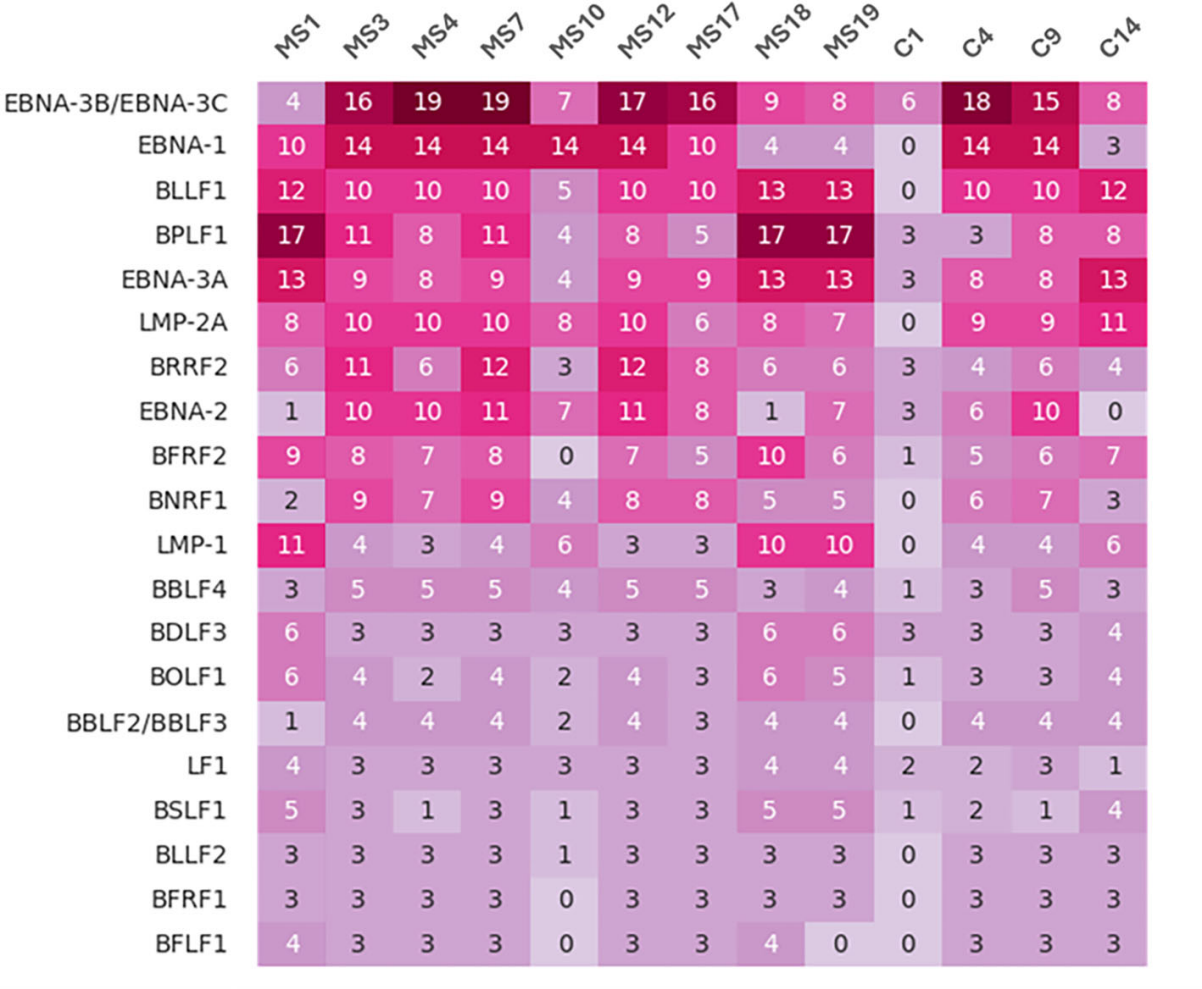
Burden analysis of missense SNVs. A heatmap containing top 20 genes with the most missense SNVs compared to reference NC007605.1.

Among missense SNVs, *EBNA-3B/C* and *EBNA-1* harbored the highest numbers of variants. Figure 3 shows the top 20 EBV genes ranked by the number of missense variants. A comprehensive summary of all missense variants per subject as well as sum of variants is provided in Supplementary Table S5.

### EBNA1 molecular mimicry region variants and HLA binding

A molecular mimicry region has been identified in EBNA1 (amino acids, aa, 377–440). This region includes motifs mimicking peptides in human proteins such as GlialCAM, CRYAB, ANO2, and MBP [7–13]. We screened the EBNA1 molecular mimicry region with margins (347–546), and found 13 variants in our cohort, 10 of which were missense. One variant (T524I) was found in all subjects with adequate sequencing coverage of the area (*n* = 12). Two variant combinations A439T–D499E; and V429M–P476Q–S492C, were 100% linked in this cohort. Among the missense variants, all 12 subjects had either A429T–D499E (two MS patients, one control) or V429M–P476Q–S492C (seven MS patients, two controls), one control lacked coverage on this region.

**Figure 4.**
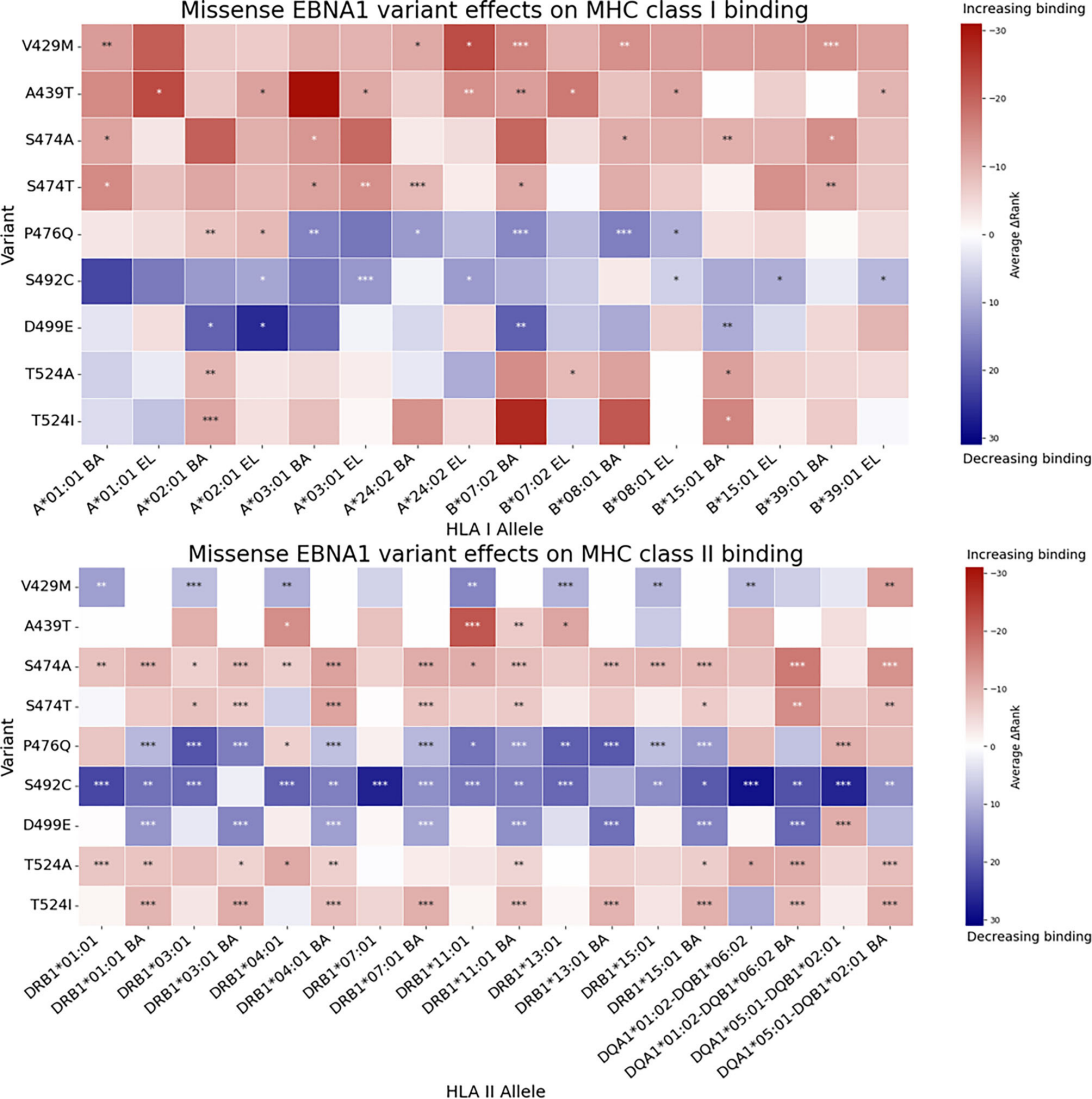
Missense EBNA1 variant effects on MHC class I and II binding. A heatmap illustrating the main findings of the EBNA1 cross-reactive region *in silico* binding analysis with common HLA alleles (*P*-value <.05 = *, *P*-value <.01 = **, *P*-value <.001 = ***; two-sided one sample *t*-test). Average rank change (ΔRank) calculated across the created EBNA1 polypeptides. Positive ΔRank (blue) means less binding potential for HLA, while negative (red) means higher binding tendency. BA = binding affinity, EL = eluted ligand. The matching EBV whole genome loci (SNV) of the variants shown are 96 946 (V429M); 96 976 (A439T); 97 081 (S474A); 97 081 (S474T); 97 088 (P476Q); 97 135 (S492C); 97 158 (D499E); 97 231 (T524A); and 97 232 (T524I).

We fragmented *in silico* the EBNA1 347–546 aa region into 15 aa (MHC II) and 12 aa (MHC I) long, overlapping peptides, generating 186 and 189 sequences respectively. We used NetMHCII-pan 4.1 and NetMHC-pan 4.1 [52, 53] to analyse the predicted changes in the HLA binding relative to the reference peptide sequences (from NC_007605.1). Average ranks in MHC binding among peptides and rank binding affinities to common HLA class I and II alleles were analysed. Nine of the variants (V429M, A439T, S474A, S747T, P476Q, S492C, D499E, T524A, and T524I) had statistically significant changes in average ranks and rank binding affinities in multiple HLA class I and II alleles (*|ΔRank|* and or *|ΔRank BA|* ≥5, two-sided one sample *t*-test, *P*-values shown in Fig. 4).

Particularly strong shifts from medium to strong binding were found in a few peptide-HLA class II allele pairs: V429M (DQA1*05:01-DQB1*02:01), S474A (DQA1*01:02-DQB1*06:02), S474T (DQA1*01:02-DQB1*06:02), P476Q (DQA1*01:02-DQB1*06:02), S492C (DRB1*04:01), and T524I (DRB1*13:01, DRB1*03:01 and DRB1*11:01). In contrast, S474T (DRB1*13:01), S492C (DRB1*15:01), D499E (DQA1*01:02-DQB1*06:02, DRB1*15:01), and S492C (DQA1*01:02-DQB1*06:02) were associated with reduced affinity (strong to medium). In HLA class I analysis, only T542I (HLA-B*07:02) caused a shift from strong to weak binding (Supplementary Table S3).

An overall increase in predicted HLA class II binding affinity was found in peptides carrying the A439T substitution (within the ANO2 mimicry core, EBNA1 aa 431–440), S474A and S474T (adjacent but not overlapping the MBP mimicry core, aa 481–498), and T524A or T524I (at the margin of the cross-reactive region). In contrast, peptides with V429M (adjacent to ANO2 mimicry core, aa 431–440), P476Q (adjacent to MBP mimicry core 481–498), S492C (within the MBP mimicry core, aa 481 – 498), and D499E (adjacent to MBP mimicry core, aa 481–498), exhibited decreased binding potential. A heatmap illustrating the main findings is shown in Fig. 4. Overall, changes in binding strength were comparable in HLA class I, with a little weaker statistical association. Detailed summaries of the EBNA1 HLA class I and II binding analysis are presented in the Supplementary Table S3.

### Haplotype analysis

**Figure 5.**
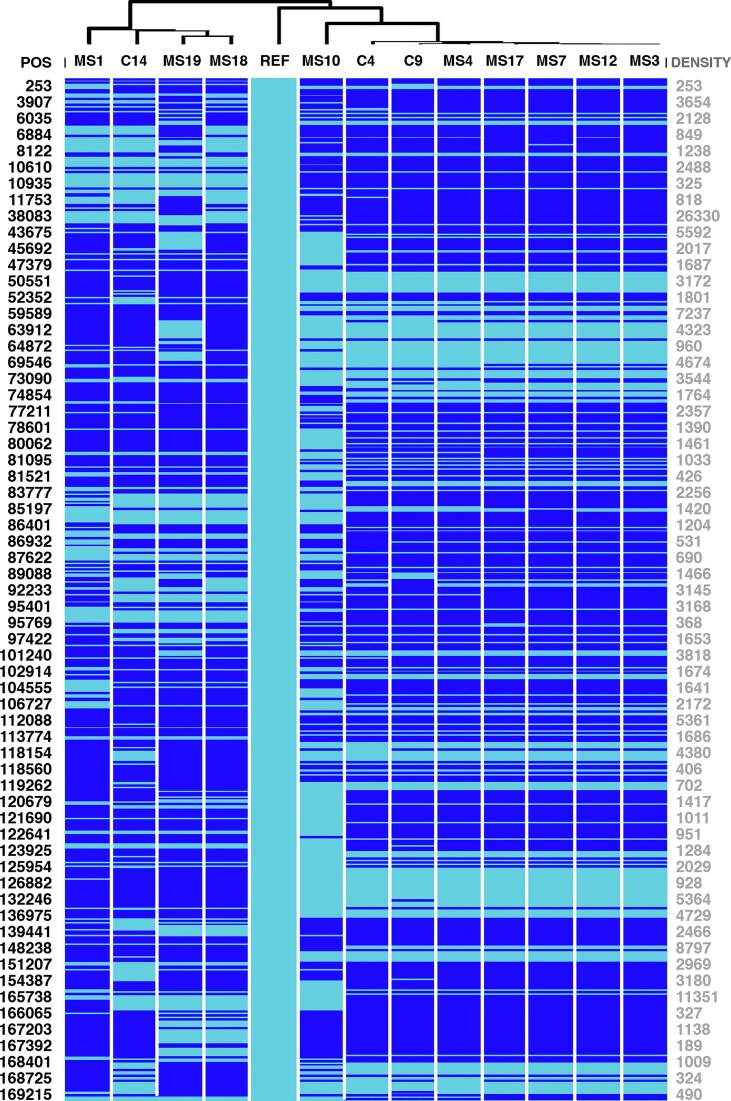
SNV haplotypes used for UPGMA phylogenetic tree construction. The haplotypes are based on 561 SNVs from positions with ≥3 read depth in all samples. Alternative alleles are coloured in dark blue and reference alleles in light blue. The genomic positions (on the left) are not shown for all markers due to image size constraints (the raw data is in Supplementary Table S5). As SNV density is not uniform across the genome, and can potentially mislead the viewer, we illustrate the density here (on the right) by subtracting the previous position from the next position on the genomic position coordinate axis. Very large difference between two coordinate positions equals a very small SNV density at the locus.

To further characterize genetic variation across the sequenced EBV genomes, we constructed an SNV-based haplotype map using 561 common SNVs found in the 13 genomes, relative to the reference (see Supplementary Table S5). Using these haplotype sequences, the EBV haplotypes clustered into three different genetic groups, best represented by the sequences of MS18, MS10, and MS3 (Fig. 5). The MS patients had viruses from all three haplotype clusters.

### Minor variant analysis

We assessed the presence of MVs, defined as SNVs with allele fractions below 50%, typically indicative of *de novo* mutations or mixed infections. Five MS cases contained MVs, the number of variants ranging from 1 to 14 (mean 6) with minor allele fraction range 4–33% (average 12%). One control had 185 MV positions, most with allele fractions between 20% and 30%. The high number of MVs and their relatively even frequency distribution suggests that this control sample had at least two co-infecting EBV strains. The low number of EBV subvariants suggest that our short culturing does not accumulate many mutations to the EBV genome. Individual distributions of MVs, are shown in Supplementary Fig. S4.

We hypothesized that shorter culturing time would result in lower accumulation of *in vitro* variants. Therefore, we compared our raw reads with LCL derived raw reads [23] from GenBank (https://www.ncbi.nlm.nih.gov/geo/query/acc.cgi?acc=GSE221624). Using Shannon entropy and two sample *t*-test, we found a significantly higher intrasample variation diversity in longer-cultured LCLs compared to our sequences, with an average per-site diversity of 0.018 versus 0.006 (*P*-value < 10^–390^; Supplementary Fig. S1). This analysis is consistent with greater accuracy of shorter culturing time, and suggests that LCL generation introduces multiple *de novo* mutations in the EBV genome, although the sequencing platform and sequencing errors (Illumina NextSeq [23] versus NovaSeq in our study) may also contribute to the diversity observed, albeit to a lesser extent.

### Multiplex-PCR analysis of selected variants

By selecting variants present in at least two of the nine MS patients and none of the four controls in regions amenable to multiplex PCR, we identified 21 SNVs. These were tested in the frozen supernatants of the 20 MS patients and 20 controls using amplicon sequencing by NGS. Out of the 21 primer pairs 15, produced the predicted amplicons in PCR (Table 2).

**Table 2.** Analysis of 15 variants of interest that were successful in multiplex PCR. Syn. = synonymous

Coordinate	Nucleotide change	Variant type (amino acid change)	MS-patients	Controls	*P*-value (Fisher’s exact)	Gene
**47 245**	A -> G	syn.(n.a.)	2/16	1/17	0.60	EBNA1, EBNA3, BFRF1
**51 848**	G -> A	syn.(n.a.)	4/18	5/20	1.00	BPLF1, EBNA1, EBNA3, BOLF1
**52 082**	G -> A	syn.(n.a.)	3/12	0/11	0.22	BPLF1, EBNA1, EBNA3, BOLF1
**70 555**	**C -> T**	**non-syn.(R -> H)**	**5/16**	**0/15**	**0.043**	**BMLF1, EBNA1, EBNA3**
**73 395**	C -> T	non-syn.(R -> Q)	4/18	2/20	0.39	EBNA1, EBNA3
**73 746**	**C -> T**	**non-syn.(R -> Q)**	**4/17**	**0/18**	**0.046**	**BSLF1, EBNA1, EBNA3**
**73 841**	G -> A	syn.(n.a.)	3/17	2/18	0.66	BSLF1, EBNA1, EBNA3
**76 567**	G -> A	premature stop(n.a.)	3/14	0/8	0.27	BLRF1, EBNA1, EBNA3
**81 061**	**G -> A**	**non-syn.(G -> E)**	**5/17**	**0/19**	**0.016**	**EBNA1, EBNA3**
**94 289**	**G -> T**	**syn.(n.a.)**	**5/18**	**0/19**	**0.020**	**BRRF2, EBNA1**
**118 154**	C -> T	non-syn.(A -> T)	5/11	6/11	1.00	BDLF1
**118 506**	G -> A	syn.(n.a.)	5/12	7/12	0.68	BDLF1
**125 389**	G -> A	non-syn.(M -> I)	10/18	13/20	0.74	BcRF1
**127 198**	G -> T	syn.(n.a.)	3/15	1/16	0.33	BcRF1
**150 471**	A -> G	syn.(n.a.)	3/18	3/20	1.00	BART, RPMS1

Nominally significant associations were found in case-control analysis at positions 70 555:C > T (BMLF1/EBNA1, *P* = 0.043), 73 746:C > T (BSLF1/EBNA1, *P* = 0.046), 81 061:G > A (EBNA1/EBNA3, *P* = 0.016), and 94 289:G > T (BRRF2/EBNA1, *P* = 0.020). While these associations did not remain statistically significant after Bonferroni correction for multiple testing (*α* = 0.0033), these and many other variants (shown in Supplementary Table S4) provide a resource for future studies. Notably, all four variants were absent in controls and are located within or near EBNA1—a viral gene central to EBV latency and immune response.

Previous findings indicate that EBV utilizes the cell’s own DNA packaging systems and monitoring (for example CTCF and PARP1) to regulate latency via 3D structural changes [58]. We analysed a subset of the sequences and high interest synonymous SNVs using an *in silico* CTCF motif predictor [59, 60]. Notably, one of the SNVs found only in MS patients (94 289:G > T [BRRF2/EBNA1]) was predicted to create a CTCF binding site, which might indicate differences in the packing and latency profile of this variant.

## Discussion

We have demonstrated that in a 6-day culture protocol, it is possible to induce endogenous EBV DNA release to the culture medium, enabling sequencing of near-complete EBV genomes from both MS patient and control blood samples. The protocol is applicable to fresh and frozen PBMCs, provided that sufficient starting material is available.

Over 1000 EBV SNVs were identified, indicating substantial variation in the EBV isolates from Finland in comparison to the reference. Albeit the number of complete MS-derived EBV sequences remains limited, our data provide foundational evidence supporting in-depth variant analysis in MS and control cohorts. Should specific EBV SNVs or haplotypes prove to be consistently enriched in MS, their effects on EBV biology could be investigated through targeted functional studies. Moreover, such variants could serve as biomarkers for disease risk stratification, and might even offer selective targets for therapeutic intervention.

Our findings include SNVs with differential frequency between MS patients and controls, including variants in noncoding regions. One MS-enriched SNV (NC007605.1:94 289:G > T in BRRF2/EBNA1) (Table 2) was predicted to create a novel CTCF binding site, suggesting potential alterations in chromatin structure and latency regulation [61]. Unstable EBV latency is pathognomonic in MS [20–23], and notably, this SNV is located relatively close (∼10 000 bp) to the EBV’s latency III promoters, Wp and Cp, which might cause sustained latency III activity. These observations align with prior studies implicating host chromatin regulators (e.g. CTCF, PARP1) in EBV latency control [58, 62]. This perspective requires additional study.

Furthermore, we identified common EBNA-1 variants in both MS patients and controls in regions of molecular mimicry. *In silico* analyses showed that these missense variants altered the predicted binding of EBNA1-derived peptides to HLA molecules. Of particular interest, the A439T substitution (located within the ANO2 cross-reactive region of EBNA1 [9]) was predicted to increase HLA class II binding affinity for all tested alleles except for HLA-DRB1*01:01 and DRB1*15:01. In contrast, V429M and P476Q, located next to the highly cross-reactive areas of ANO2 and MBP, respectively, were associated with decrease in HLA binding affinity. Such reductions could potentially favour the presentation of peptides from neighbouring, more cross-reactive regions of EBNA1. All subjects with sequence coverage at these variants’ loci, carried either the A439T-D499E (*n* = 3) or the V429M-P476Q-S492C (*n* = 9) combinations, which makes them interesting targets for further studies.

A technical limitation of hybrid capture methods is the potentially incomplete representation of genomic regions, particularly when probe sequences do not sufficiently match divergent viral strains. This could introduce bias or lead to underrepresentation of certain loci. To address this, our probe set was designed using EBV genomes from multiple strains, representing diverse geographic and genetic backgrounds, thereby improving the likelihood of comprehensive capture across different viral lineages. As shown in Fig. 1, an uneven representation of the EBV genome was obtained in the current probe set and further refinement of probe design, and inclusion of newly sequenced strains, in particular from healthy individuals, could help mitigate bias and enhance coverage uniformity. All SNVs were identified relative to the EBV reference genome NC_007605.1, constructed using the EBV B95-8 sequence as backbone, and complemented with the Raji sequence. These strains originate from geographically and genetically distinct, non-MS sources. Future studies should prioritize the establishment of region- and disease-specific reference genomes or the application of pan-genomic analysis to more accurately distinguish background variation from putative disease-associated mutations.

To ensure the reliability of the SNVs detected, we applied stringent filters during variant calling, including a minimum read depth of 3, an allele frequency cutoff at 20%, a mapping quality of >20, an incorrect call probability threshold <10^–6^, and strand bias <10^–5^. Importantly, SNVs were only called in regions with consistent coverage and excluded if supported by low-confidence reads or located in homopolymer-rich or repetitive regions prone to sequencing errors. To minimize culture-related variations and selection artefacts, we employed a short, 6-day induction protocol in which EBV release began during the final 24 h. This approach increases the likelihood that the detected variants reflect the *in vivo* EBV genetic landscape rather than *in vitro* adaptation. Supporting this, 76% of the variants were shared between sequences from *ex vivo* CD19^+^ cells and culture supernatants, 94% of the 1088 variants were detected in three or more donors, MVs were rare, and the mean per-site Shannon entropy was low (∼0.006).

To our knowledge, this is the first report of complete, noncancer-derived EBV sequences obtained without extended *in vitro* LCL propagation both in MS patients and healthy controls. Access to full-length sequences enables comprehensive characterization of the viral properties, and information for EBV vaccine development [63]. Given the inherent contribution of EBV in MS pathogenesis, understanding of EBV biology and its antigenic structures is crucial for uncovering its role in disease. At least four mechanistic scenarios can be envisioned: (i) EBV variants may destabilize latency [20, 22, 23, 64]. We identified variants in several latency regulating genes, including EBNA1, LMP-1, LMP-2B, EBNA2, BHRF1, and EBNA-3A, which could promote repeated viral reactivations and spread. LMP-1 and -2 can also hijack host cell’s signalling pathways by mimicking CD40 and B cell receptor signalling. (ii) EBV variants may change interactions with B cell chromatin, leading to aberrant transcription and B cell functions [65–67]. We detected several variants in chromatin modulating genes, such as BZLF1, BRLF1, EBNA2, and EBNA-3B/C. (iii) Nonsynonymous variants may generate novel antigenic epitopes; we found 355 such variants, which could potentially lead to new epitopes. Indeed, several SNVs were discovered in the molecular mimicry region of EBNA1, and were predicted to alter peptide binding to HLA. (iv) Variants may facilitate viral entry into B cells or expand tropism to other cell types [68]. We found 13 missense variants in BLLF1 (encoding gp350/220 used to infect B cells), 2 missense variants in BZLF2 (encoding gp42 that binds MHC class II on B cells), 3 missense variants in BXLF2 or BKRF2 (encoding gH/gL complex that interacts with receptors on epithelial cells and B cells) and one missense variant in BMRF2 (that binds integrins on epithelial cells). These variants and their frequencies are shown in Supplementary Tables S4 and S5. Together, these findings suggest that EBV genetic diversity may contribute to MS pathogenesis through multiple mechanisms.

In conclusion, we developed a novel method for sequencing endogenous EBV genomes from blood samples of MS patients and controls, demonstrating high degree of EBV sequence variation and offered preliminary evidence of differences between the groups and possible MS associated EBV variants. Our dataset provides a valuable resource for further studies and the methodology, a foundation for large-scale analysis of EBV diversity in MS and other EBV-associated diseases.

## Supplementary Material

ugag019_Supplemental_Files

## Data Availability

All the sequencing data of the project are made available on request. All other data are available in the main text or the supplementary materials. The code of TRACESPipe can be found in Zenodo (DOI:10.5281/zenodo.7646369). The sequences with breadth coverages over 90% have been submitted to GenBank with accession numbers PQ559301–PQ559312; numbers PQ559301–PQ559309 for MS patient derived EBV genomes, PQ559310–PQ559312 for controls.
