# Supplementary material for: EBV genome analysis in multiple sclerosis shows extensive viral diversity and links to autoimmunity
Source: NAR Mol Med. 2026 Mar 24;3(2):ugag019. doi: 10.1093/narmme/ugag019 (PMC13064507; doi:10.1093/narmme/ugag019)

Supplementary Figure S1. Shannon Entropy per site comparison. We used units in bits (log_2) and two sample T-test to address intrasample variation diversity from all possible sources. (*** = p-value < 10^{-390}).

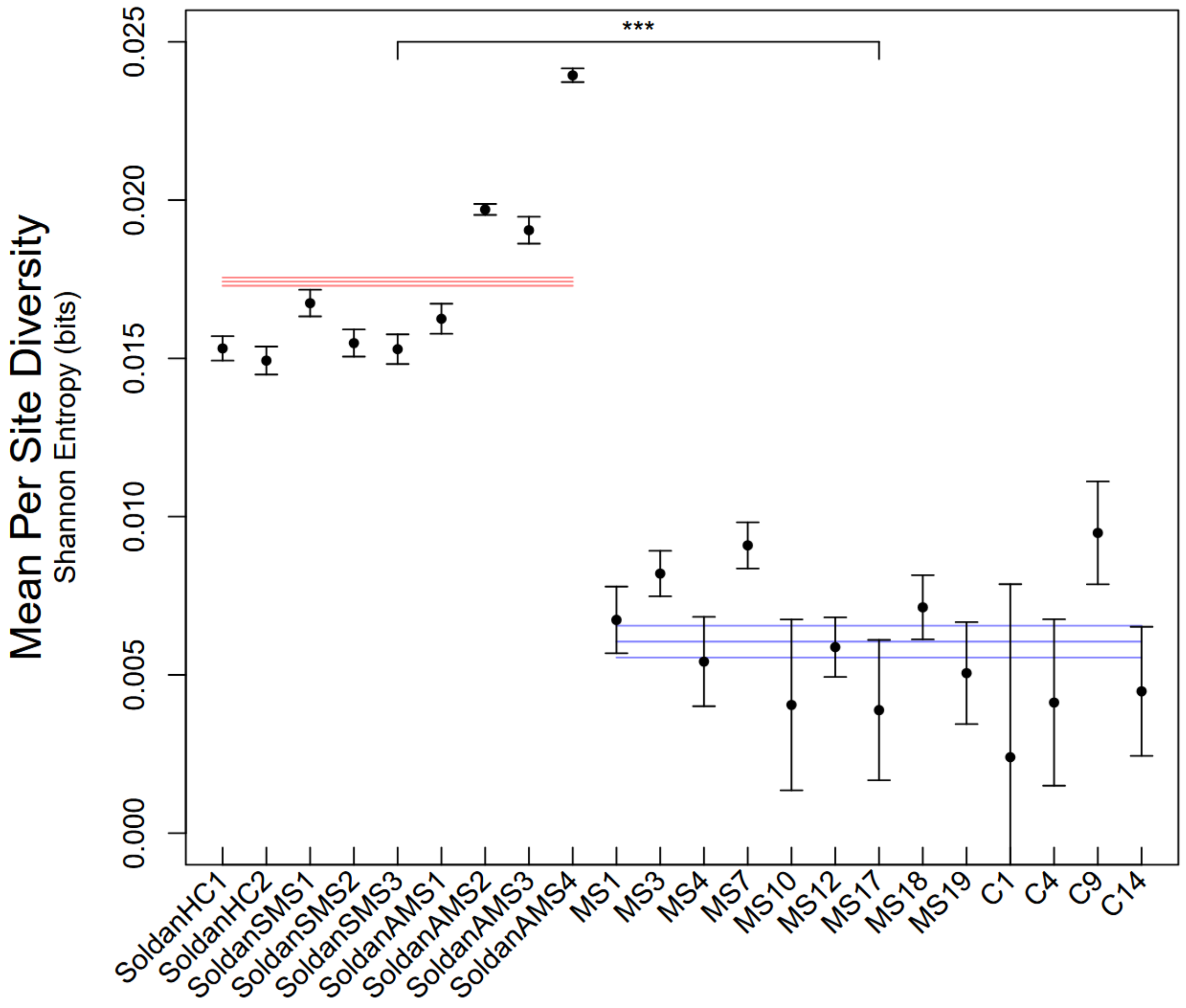

Supplementary Figure S2. Overview of the protocol for endogenous EBV enrichment.

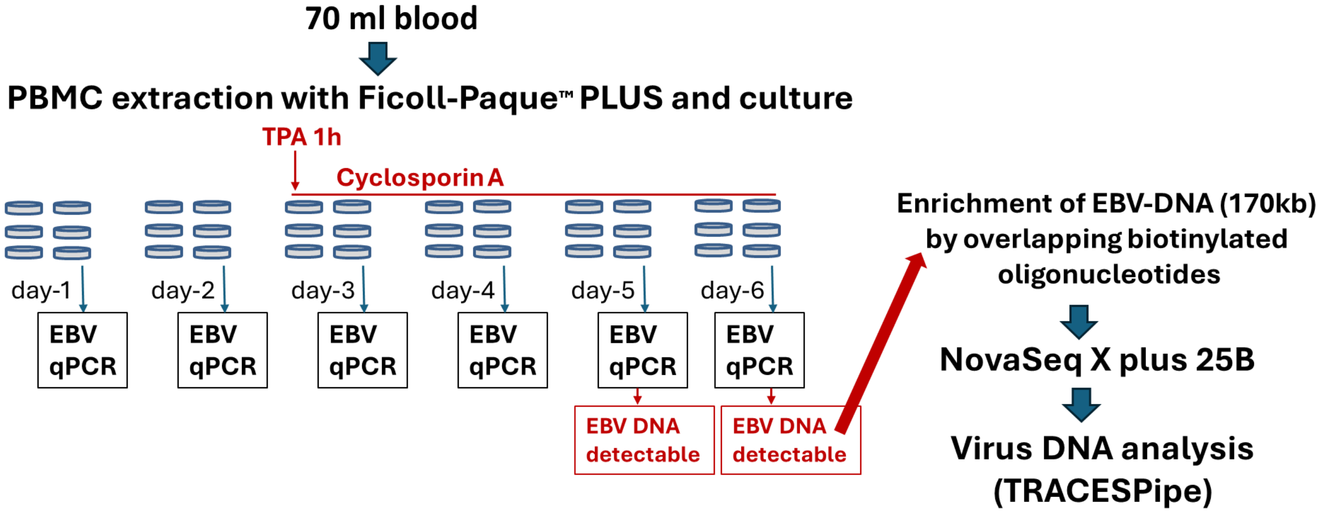

Supplementary Figure S3. SNV Distance Matrix. Number of SNVs in pair-wise comparisons of the reference (NC_007605.1), MS1-19 (n=9) and control (C1-14, n=4) sequences. SNVs filtered with variant call quality of >20, mapping quality of >20, and read depth >3, repeat regions excluded.

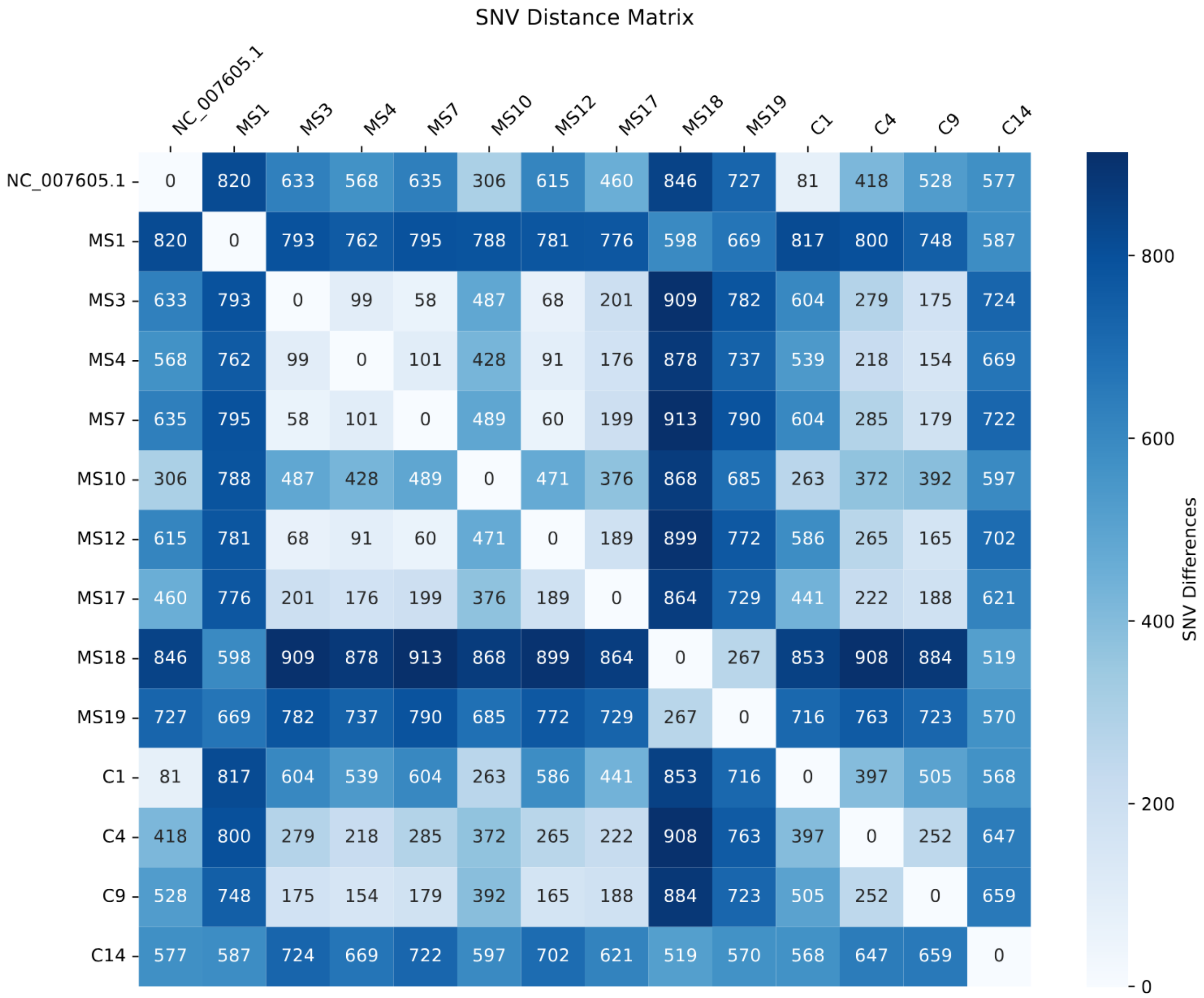

Supplementary Figure S4. Individual EBV minor variant distribution. Minor variants, their frequencies and types illustrated. Minor variants were found from 5 MS patients and one control, who most likely has two competing EBV strains.

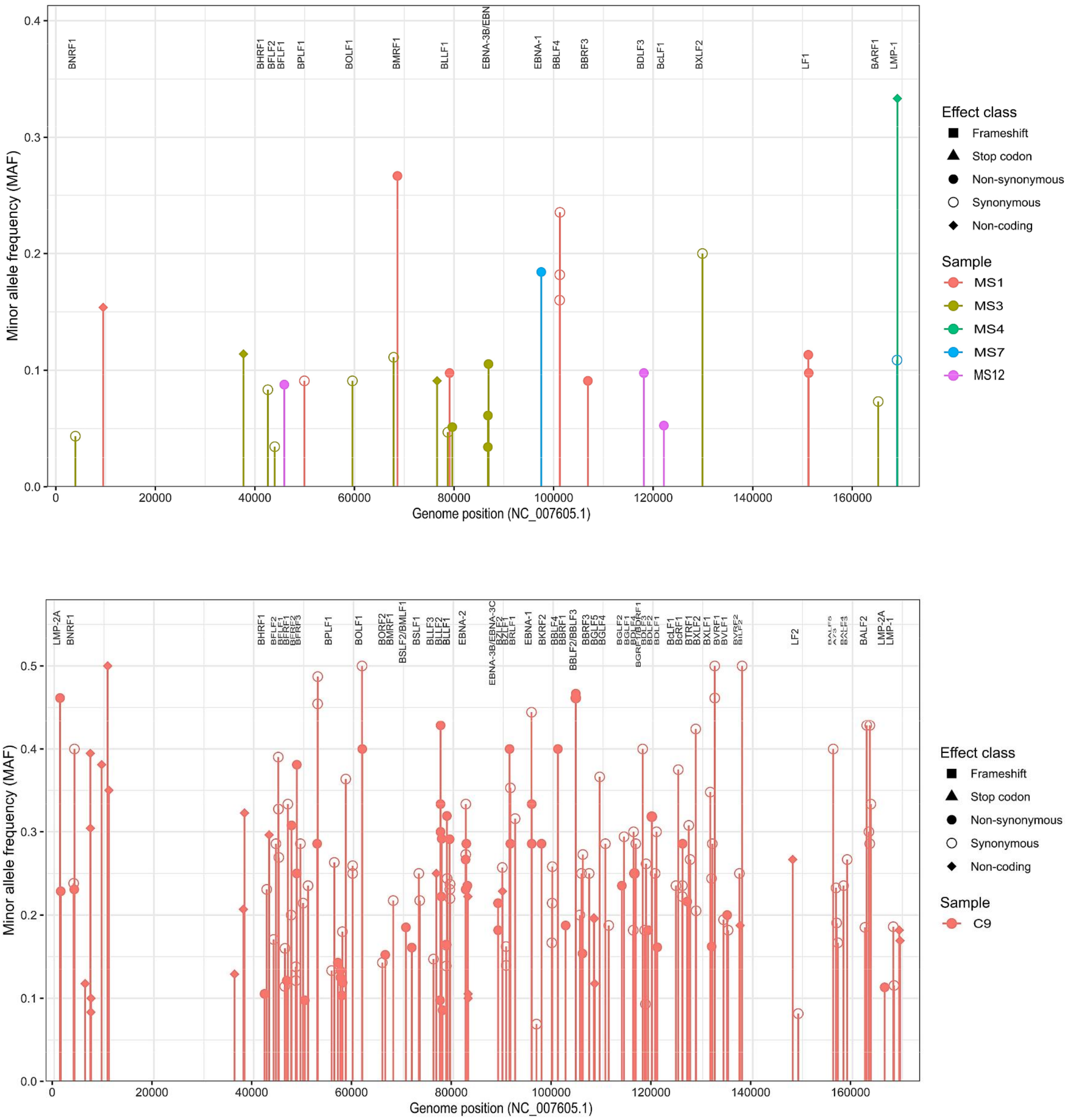

Supplement: ugag019_Supplemental_Files [file ugag019_supplemental_files.zip › Supplemetary_Figures_S1_S4_Combined_NAR_Molmed.pdf]
